# Localization of single molecules with structured illumination and structured detection

**DOI:** 10.1038/s41377-025-01980-1

**Published:** 2025-09-29

**Authors:** Luciano A. Masullo

**Affiliations:** Max Planck Institute of Biochemistry, Planegg, 81252 Germany

**Keywords:** Super-resolution microscopy, Confocal microscopy

## Abstract

A new high-precision single-molecule localization scheme, ISM-FLUX, is an implementation of MINFLUX using image-scanning microscopy (ISM) with a single-photon avalanche diode (SPAD) array detector. ISM-FLUX results in a larger localization range, enhancing the robustness of the localization scheme and it also potentially enables experiments in which absorption and emission of a single fluorophore can be probed independently.

Precise localization of individual fluorescent molecules is essential for physicochemical and biophysical techniques, including single-molecule tracking^1,2^ and super-resolution imaging using single-molecule localization microscopy^3–5^.

Most common single-molecule tracking and single-molecule localization microscopy (SMLM) techniques, such as photoactivated localization microscopy (PALM)^3^, stochastic optical reconstruction microscopy (STORM)^4^, and points accumulation for imaging in nanoscale topography (PAINT)^5^, are based on wide-field imaging setups. In these methods, a relatively large area of the sample (hundreds of μm²) is uniformly illuminated by a laser, and the image of the fluorescent molecules is recorded in a very sensitive scientific camera. The low density of (active) fluorescent emitters at a given point in time allows for their precise localization in each camera frame, enabling the reconstruction of a super-resolved image—or a trajectory, in the case of single-molecule tracking. The information about the position of the fluorescent molecules is retrieved from the detected signal, thanks to the structured nature of the detector, i.e., the array of pixels. This group of methods localizes single molecules using a structured detection and a uniform, constant illumination.

Over the past two decades, alternative approaches have emerged that localize single emitters not by analyzing their camera-captured images, but by detecting photon counts with a point-like detector (e.g., a photodiode) as the emitter is illuminated with a sequence of spatially structured light patterns^6–8^ (see ref. ^9^ for an in-depth discussion of these methods). The emitter position is then estimated by comparing the relative photon counts corresponding to each illumination step, together with the a priori knowledge of their spatial structure and pattern. Among these, MINFLUX^10^ is particularly noteworthy, offering nearly tenfold higher localization precision compared to conventional wide-field, camera-based methods, achieving ~1–2 nm precision with moderate photon counts (~1000) by sequentially illuminating the sample with a light pattern featuring an intensity minimum.

Recently, Radmacher et al.^11^ integrated sequential structured illumination with structured detection, combining image scanning microscopy (ISM) with single-molecule localization microscopy (SMLM), and achieved up to a twofold improvement in localization precision. In their recent work in *Light: Science & Applications*^12^, Slenders et al. experimentally realize ISM-FLUX^13^ by combining a sequential structured illumination featuring a minimum of intensity (Fig. 1a) with a structured detection (Fig. 1b). The experimental approach is based on a confocal scheme in which they use standard galvanometric mirrors (as done in RASTMIN^14^) to scan a doughnut-shaped beam in a circular pattern and replace a point-like detector with a single-photon avalanche diode (SPAD) array (Fig. 1c). The advantages of this new method are several: (i) enhanced range and robustness for tracking of single molecules, (ii) simplified and more versatile architecture, (iii) enabling correlative localization of the absorption and emission of the single emitter.

(i) A constraint of MINFLUX is that the molecule to be localized must reside within the target coordinate pattern (TCP) of illumination light (≤200 nm). If the molecule is outside the TCP, its position cannot always be uniquely determined from the data, and the tracked molecule can be lost. Harvesting the extra information provided by the array detector and the image of the molecule recorded, ISM-FLUX provides good localization precision and accuracy within an extended region of up to 600 × 600 nm^2^.
  State-of-the-art widefield super-resolution methods, such as latest implementations of DNA-PAINT^15,16^, routinely achieve localization precisions of ~2 nm, and the recently developed RESI^17^ achieves Ångström resolution in intact cells^18^ over ~100 × 100 μm^2^ fields of view, surpassing the performance of MINFLUX both in resolution and throughput using significantly simpler optical setups. However, MINFLUX and related methods have a huge advantage in live-cell applications^19–21^: the photon-efficient (and thus time-efficient) localization schemes are ideal to follow trajectories of biomolecules in living systems where, unlike in fixed cells, the photon efficiency and time resolution play a key role in practical performance. In this context, the enhanced localization range of ISM-FLUX provided by the structured detection will enable more robust and accurate single-molecule tracking measurements, boosting MINFLUX applications in living cells.
(ii) ISM-FLUX is implemented in a confocal microscope with a conventional scanner using galvanometric mirrors, the TCP can be easily calibrated using the structured detection, and, importantly, the setup is based on open-source efforts^22–24^. Despite the significant impact of MINFLUX, its replication has been limited beyond the inventor’s lab and the commercial implementation. By complementing ongoing efforts in open-source hardware^24–27^ and software^9,28,29^, ISM-FLUX has the potential to make this advanced technology accessible to a wider community of microscopy developers and users, thus maximizing the development of the methods and expanding the applications.
(iii) Finally, ISM-FLUX (and also ISM-SMLM^11^) could provide unique insight into complex light-matter interaction phenomena where light absorption is spatially decoupled from light emission at the molecular level, such as π-conjugated polymers^30^, H and J-type aggregates^31^, and light-harvesting complexes (LHC)^32^. By comparing the sequential structured illumination and the structured detection measurements (instead of combining them), ISM-FLUX could independently probe the spatial location of light absorption and light emission of the same molecule. Importantly, ISM-FLUX maintains single-photon counting capabilities in its detection, thus enabling complementary spectroscopic measurements such as fluorescence lifetime and photon (anti)bunching. On the other hand, simultaneous and independent probing of absorption and emission enables cross-calibration of techniques like MINFLUX (sequential structured illumination) and RESI (structured detection), allowing direct comparison of their accuracy and precision within the same measurement. This approach could, for example, directly test the hypothesis of freely rotating dyes.

**Fig. 1:**
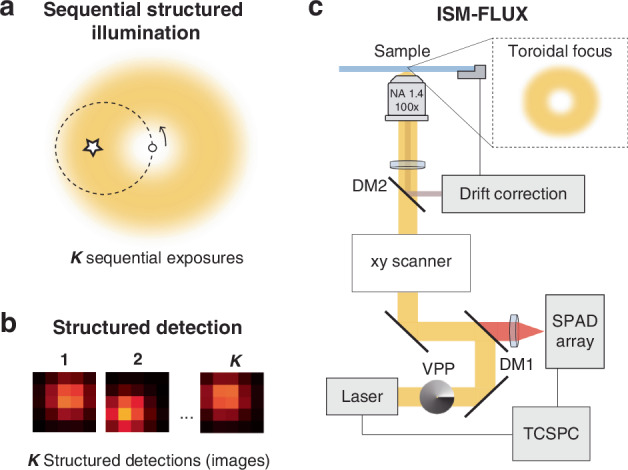
Schematic of ISM-FLUX. **a** Sequential structured illumination with a toroidal beam in ISM-FLUX, **b** Structured detection corresponding to each of the sequential exposures. **c** Schematics of the experimental setup. Based on a standard confocal microscope, ISM-FLUX includes a vortex-phase plate (VPP) in the excitation path to generate a toroidal (doughnut-shaped) focus, and a SPAD array in the detection that is connected to a time-correlated single-photon counting (TCSPC) unit to perform spectroscopic measurements

In summary, ISM-FLUX has exciting potential to boost the performance of state-of-the-art single-molecule tracking experiments in living cells, expand the incorporation and development of MINFLUX-like technologies, and provide insights into complex light-matter interactions at the molecular scale.
